# Carbon Nanotube Based Groundwater Remediation: The Case of Trichloroethylene

**DOI:** 10.3390/molecules21070953

**Published:** 2016-07-21

**Authors:** Kshitij C. Jha, Zhuonan Liu, Hema Vijwani, Mallikarjuna Nadagouda, Sharmila M. Mukhopadhyay, Mesfin Tsige

**Affiliations:** 1 Department of Polymer Science, The University of Akron, Akron, OH 44325, USA; zl23@zips.uakron.edu; 2 Center for Nanoscale Multifunctional Materials, Wright State University, Dayton, OH 45435, USA; vijwani.2@wright.edu (H.V.); nadagouda.mallikarjuna@epa.gov (M.N.); sharmila.mukhopadhyay@wright.edu (S.M.M.)

**Keywords:** carbon nanotubes, groundwater remediation, VOC removal, trichloroethylene, CNT hybrid, CNT membrane, hierarchical catalysis, biomimetic

## Abstract

Adsorption of chlorinated organic contaminants (COCs) on carbon nanotubes (CNTs) has been gaining ground as a remedial platform for groundwater treatment. Applications depend on our mechanistic understanding of COC adsorption on CNTs. This paper lays out the nature of competing interactions at play in hybrid, membrane, and pure CNT based systems and presents results with the perspective of existing gaps in design strategies. First, current remediation approaches to trichloroethylene (TCE), the most ubiquitous of the COCs, is presented along with examination of forces contributing to adsorption of analogous contaminants at the molecular level. Second, we present results on TCE adsorption and remediation on pure and hybrid CNT systems with a stress on the specific nature of substrate and molecular architecture that would contribute to competitive adsorption. The delineation of intermolecular interactions that contribute to efficient remediation is needed for custom, scalable field design of purification systems for a wide range of contaminants.

## 1. Introduction

**Figure 1:**
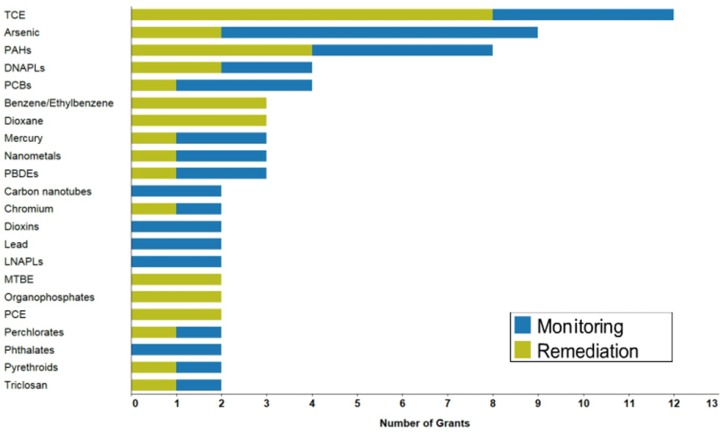
Relative share of contaminants studied by projects funded through the Superfund Research Program (FY 2011–2015). Reprinted with permission from National Institute of Environmental Health Sciences (NIEHS).

Groundwater contamination is an ever present danger with the introduction and detection of new chemicals that have been demonstrated as toxic in shallow aquifers and wells through evolving industrial processes. Among the organic contaminants, due to its widespread use in spray, varnish, and dry cleaning industries, trichloroethylene (TCE) is a major groundwater pollutant [1,2,3,4,5,6]. The solubility of TCE in water, its volatility, and lack of standard disposal practices have made it the focus of remediation and monitoring studies [7,8,9,10,11,12,13] (Figure 1). In dosage exceeding 250 ppm, TCE is neurotoxic and is classified as “probably carcinogenic in humans” by the International Agency for Research on Cancer [14].

Approaches for removal, degradation and detection of TCE have commonly included activated carbon [15,16,17,18,19,20,21,22,23] as a host and adsorbent. Even though activated carbon and naturally sourced materials such as pine needle biochar [24,25] have orders of magnitude advantages in cost, the use of two-dimensional carbon based materials (graphene nanosheets, graphene oxides and various carbon nanotubes (CNTs) with different chiralities) offers specificity in adsorption and remediation with relatively lower % loading in membranes and composites. CNTs have continued to drop in price and are now acceptable as applied materials in scalable processes including water purification [26,27,28,29]. A large number of approaches that incorporate CNTs in hybrid and hierarchical systems take advantage of the aspect ratio and specific adsorption through surface functionalization. We discuss some of these approaches in the following sections and provide context in terms of the effect of hierarchy, catalytic availability, mobility, and specific adsorption. Section 2 is an examination of current strategies, while Section 3 and Section 4 present experimental and modeling results based on the perspective offered by Section 2.

## 2. Strategies for TCE Removal: CNT Membranes and Hybrids

**Figure 2:**
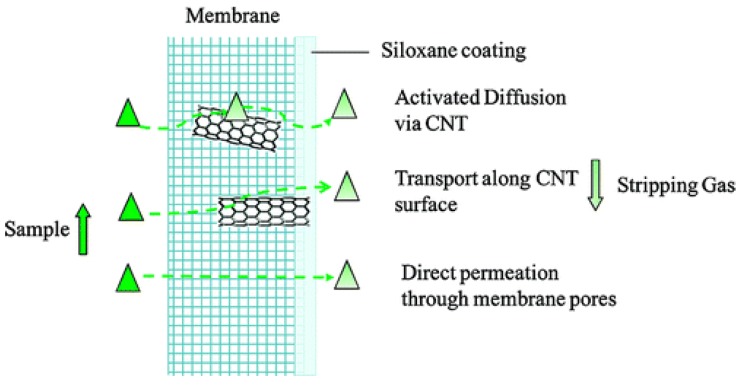
Effect of carbon nanotubes (CNTs) in membrane based pervaporation process. Analytes are represented by triangles. Reprinted with permission from Ref. [30]. ©2010 American Chemical Society.

The high strength and aspect ratio of CNTs has led to their incorporation in composite tmembranes for removal of organic contaminants, with improved efficiency and mechanical strength [30,31,32,33,34,35,36,37,38]. Additionally, the flux of composite membrane materials was also found to increase for a polydimethylsiloxane (PDMS) matrix with CNT fillers, which may be ascribed to the water slip behavior on nanotubes reported by molecular dynamics simulations [39]. Incorporation of CNTs in polyvinylidene difluoride (PVDF) hollow fiber membranes, commonly used for pervaporation, resulted in enhancement of TCE remediation by 27 % at 303 K. The combined effects of high surface area and smooth, frictionless exterior lead to changes in diffusion, flux, and adsorption in the composite membrane as shown in Figure 2 [30].

**Figure 3:**
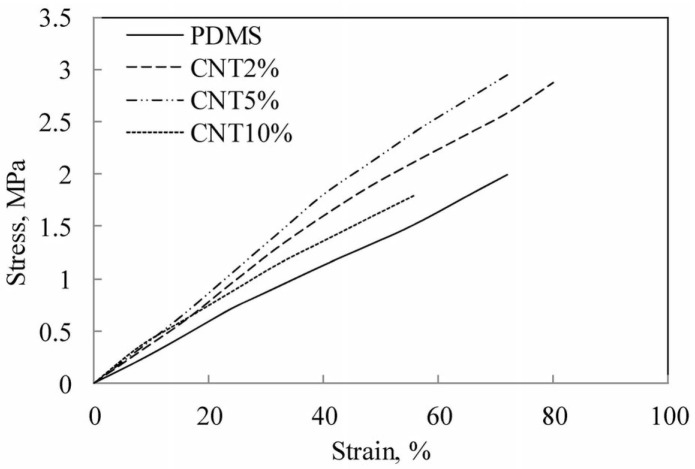
Stress strain curves with CNT loading for polydimethylsiloxane(PDMS) matrix membranes. Reprinted with permission from Ref. [40]. ©2014 Nature Publishing Group.

The strength of composite membranes increases with CNT loading (Figure 3). For a PDMS matrix, it was found that the modulus increases up to 10% loading of CNT after which there is a decrease, but the modulus still remains higher than the original PDMS matrix. For a poly(acrylic acid) (PAA)/polyvinyl alcohol(PVA) crosslinked nanofibrous matrix with zerovalent iron, there was an increase of up to 62.6% in modulus upon addition of multiwall carbon nanotubes (MWCNTs) [36].

**Figure 4:**
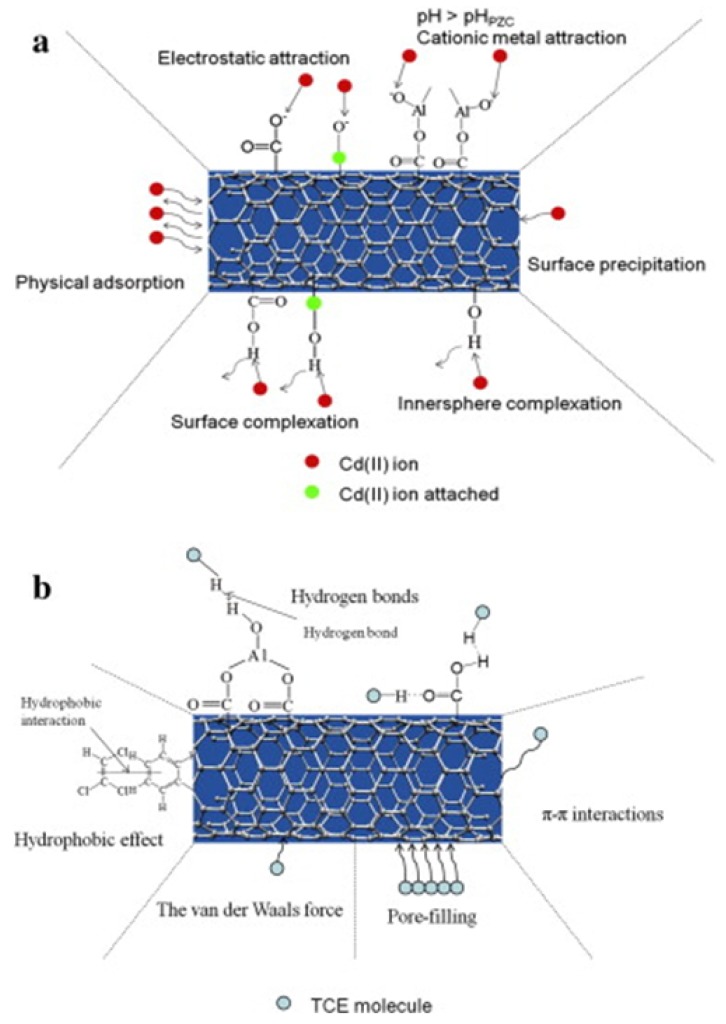
Interaction of alumina coated MWCNTs with (**a**) Cd(II) ion, and (**b**) TCE. The schematic shows the range of interactions that contribute to adsorption processes in hybrid, hierarchical systems. Reprinted with permission from Ref. [41]. ©2015 Elsevier.

Metal oxides have been frequently used in conjunction with CNTs and offer two advantages. First, they can aid in adsorption, such as the coating of aluminium oxide on CNTs that resulted in a multifunctional membrane that can remove heavy metal (Cd(II)) ions as well as TCE through preferential electrostatic affinity [41]. Second, they produce OH radicals that can be used in advanced oxidation processes, which are Fenton like reactions that take place in a broad range of pH values [42,43]. Such versatility means choice of CNTs and metal oxides can produce a combinatorial solution to specific effluent remediation. The case of aluminium oxide, and the resulting interactions that contribute to remediation are shown in Figure 4.

### 2.1. CNT Interactions with Matrix and COCs

Functionality through incorporation of CNTs in a matrix can be viewed as CNTs acting as active sorption sites. Their surface physical properties, through functionalization, would guide the flow (hence the flux) of the analyte as well as its non-covalent and covalent interactions with the CNT. A pristine CNT may increase the flux and, depending on molecular structure, also be selective to an analyte. If the CNT is functionalized, with alumina for example (Figure 4), then electrostatic interactions may dominate the adsorption of an electronically rich part of the effluent (such as heavy metal), while the hydrophobic exposed part of the CNT interacts through van der Waals and soft epitaxial interactions.

**Figure 5:**
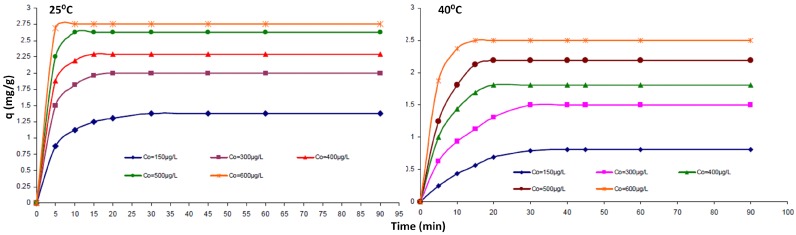
Adsorption isotherm of TCE onto MWCNTs for two diffferent temperatures. Reprinted with permission from [46].

For the specific case of MWCNT–TCE interactions, there was a decrease in adsorption of TCE with temperature as shown in Figure 5. This points to a mechanism of physisorption, which was also observed by Tanada et al. [44] for activated carbon fiber. In the case of alumina coated CNTs, the change in pH does not affect the adsorption of TCE, while it has a marked effect on the adsorption kinetics of Cd(II) [41], also pointing to a physisorption based mechanism for TCE. Physisorption would be guided by molecular matching to the substrate (soft epitaxy) and change in planarity of the particular molecule (or COC) would change the non-covalent interactions that determine such adsorption.

**Figure 6:**
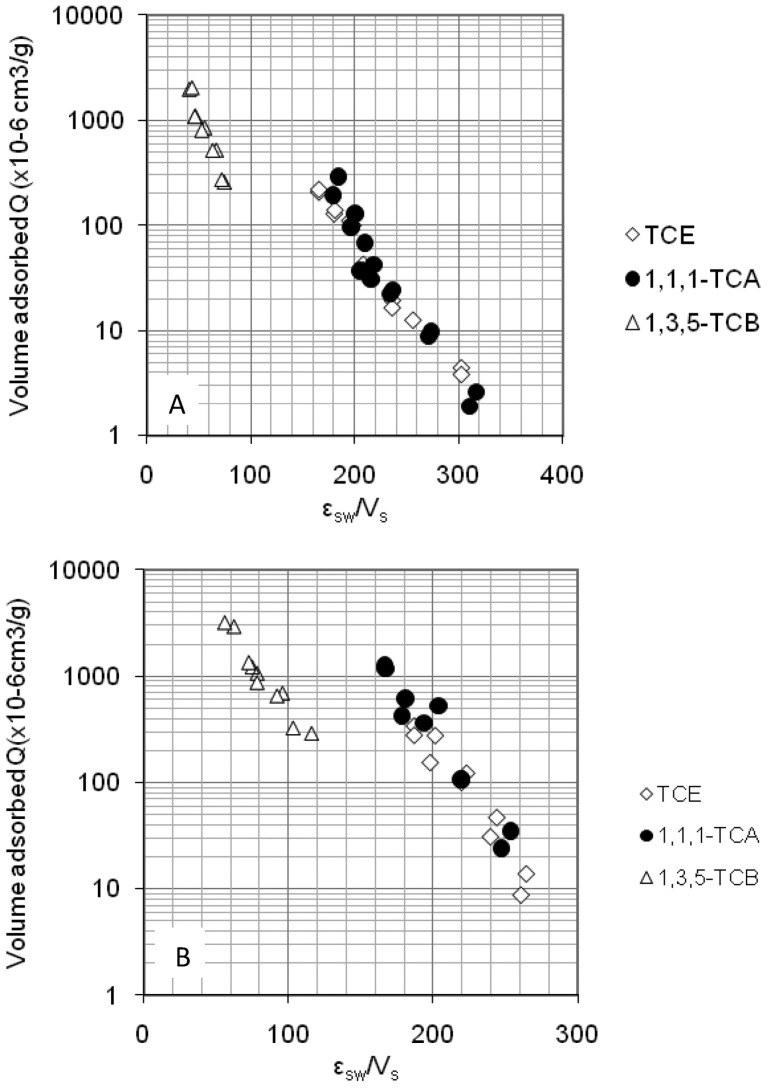
Correction curves for pristine (**top**) and heat treated (**bottom**) MWCNTs for adsorption of TCE, 1,1,1-1, 1, 1-trichloroethane(TCA) , and 1,3,5-trichlorobenzene TCB. Reprinted with permission from [45]. ©2015 American Chemical Society.

A systematic study of three different COCs on MWCNTs by Ma et al. [45] showed that correction curves, normalized by molecular volume, did not yield sorption isotherms that coalesced, as should be expected through fitting the isotherms using the Polanyi-Manes Model (PMM) (Figure 6) [46]. They hypothesize that electron–donor interactions, for the case of 1,3,5-trichlorobenzene (1,3,5-TCB), was the cause of deviation. Polarizability, hydrogen bond interaction, and electron–donor interaction would all contribute to the characteristics of sorption isotherms in addition to molecular volume. These particular intermolecular forces would vary based on the chirality and pore size of the CNT.

**Figure 7:**
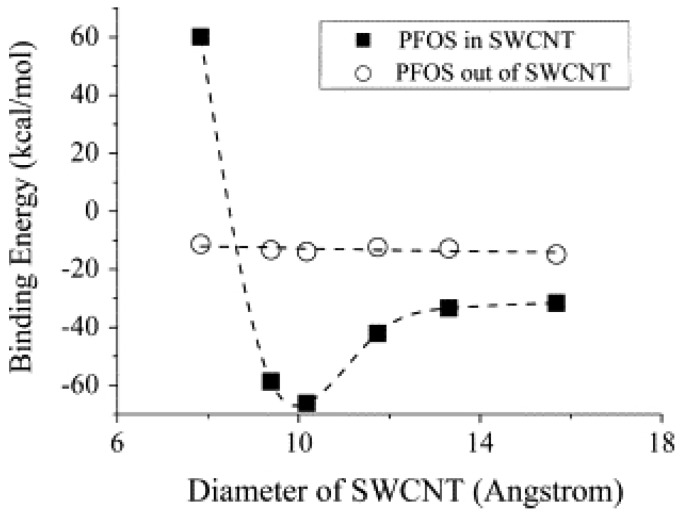
Diameter dependence on binding energies of perfluorooctane sulfonate (PFOS), inside and outside of SWCNTs. Reprinted with permission from [47]. ©2013 Elsevier.

One particular method to mine these intermolecular forces would be to tune the CNT diameter. The dependence of COC adsorption on CNT diameter is an under-explored area where curvature effects, wall repulsion, and cohesive packing would contribute to specific variance for a molecule. It has been shown that with increase in diameter of CNT, there is an increase in adsorption of perfluorooctane sulfonate (PFOS) to the inner layer of the CNT upto a certain point, after which the trend reverses and saturates [47] (Figure 7). For diameters below 1 nm, the repulsive interactions between PFOS molecules is high, contributing to the large adsorption energy. As the diameter increases, there is a balance between wall repulsion and cohesive interactions between PFOS molecules. On the outer side of the CNT, the PFOS is free from confinement effects and can interact with the CNT surface with relatively smaller changes in deformation and displacement. Since the adsorption process with variance in CNT diameter is highly dependent on molecular achitecture, in a mixture of COCs, the substrate specific competitive adsorption effects would determine remediation.

### 2.2. Competitive Adsorption of COCs

The number of pores for a given matrix-CNT composite are fixed. This leads to competitive adsorption effects that take place in effluent mixtures, most commonly in the presence of natural organic matter (NOM) [22,48,49,50,51,52].

In a recent study, Ersan et al. [49] found that the adsorption of both TCE and phenanthrene (PNT) decreased on a number of two-dimensional graphene materials, including CNTs, by the presence of background organic matter due to competition in site sorption or pore blockage. At the same time, the decrease in PNT sorption was more than the comparative decrease in TCE sorption, because of the larger planarity of PNT and its ability to interact via *π*-*π* stacking which would be disrupted, to a greater extent, through decrease in surface size and availability.

In a separate study that uses bacterial immobilization on hierarchical CNT structures, it was found that a high level of sensitivity could be obtained for all background materials, except when TCE was competing with toluene [53]. The interference provided by a particular contaminant would be specific to the architecture and the nature of the active material hosted on CNTs (viz. zero valent ions, bimetallic NPs, bacterial cultures). In most cases of detection and remediation, it would be a superposition between the effects of active material-contaminant and host (CNT)-contaminant–interactions. Active-material–contaminant interactions can be isolated through in situ studies (for example titania or a bacterial culture with a host of contaminants), while the nature of the complex hierarchy contributions are less clear. This is the focus of our next section on hierarchical effects.

## 3. CNT Hierarchical Hybrid Approach for TCE Remediation

Use of hierarchical hybrids for enhanced catalytic activity and water filtration has gained increasing traction over the past few years [54,55,56,57]. Distribution of CNTs in a matrix and the overall morphology would determine the efficiency of purification/catalysis and a few biomimetic approaches take advantage of hierarchies that are known to be potent in nature [56,58,59,60,61,62,63,64].

**Figure 8:**
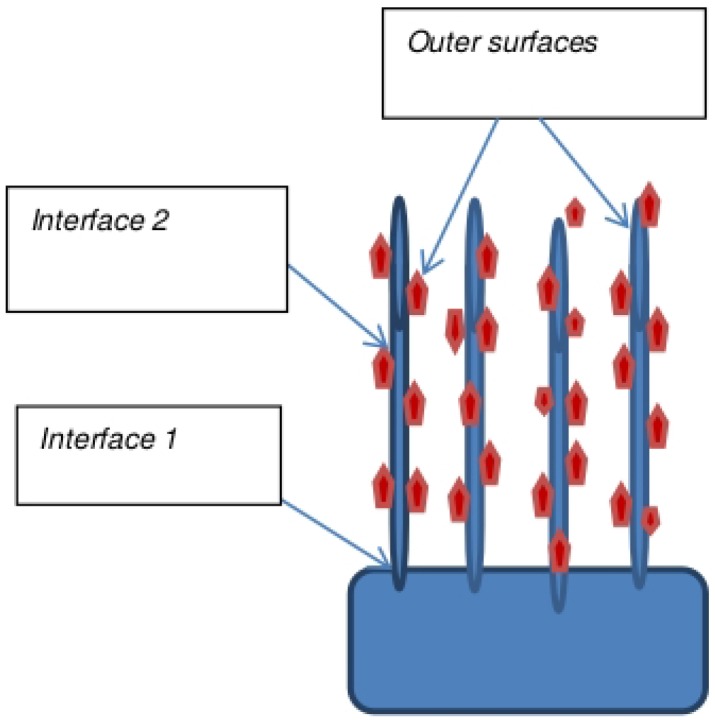
Hierarchical design with the increased number of interfaces. Interface 1 is between the CNT and porous carbon substrate and Interface 2 is between CNT and nanoparticles.

**Figure 9:**
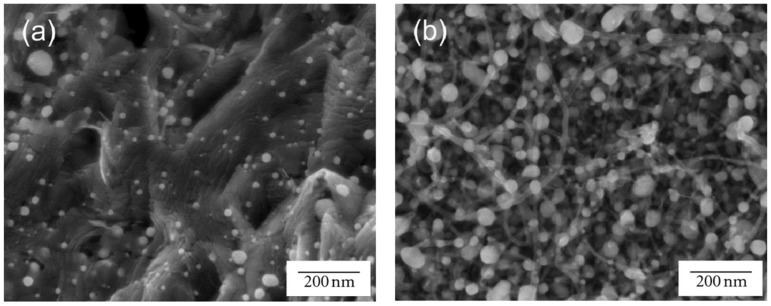
SEM micrographs of two different hierarchies: (**a**) Palladium nanoparticles (Pd–NPs) supported on carbon foam, and (**b**) Pd–NPs supported on CNT-grafted carbon foam. Reprinted with permission from [65]. ©2012 Hindawi Publishing Corporation.

To obtain large increments in remediation, a smart hierarchical approach that increases the surface area for remediation, while also dispersing catalytic particles for highest availability, is proposed by Mukhopadhyay et al. [60,61,62,63,64,65,66] (Figure 8 shows the schematic and Figure 9 shows the SEM micrographs). CNT forests are fabricated onto a porous carbon substrate, with a dispersion of nanoparticles that is controlled by surface functionalization. The resulting morphology has a large number of nanoreactors where the fluid flow leads to enhanced remediation.

It should be noted that nanotubes [67] and graphene [68] have been known to disperse in chloroalkanes. The grafting and functionalization of CNTs in the current architecture, as well as the low levels of COC concentration compared to pure solvents, would preclude such activity. If the nanotubes are not grafted and the COC concentration is high, dispersion would have to be accounted for in the fabrication of the hybrid remediation architectures.

**Figure 10:**
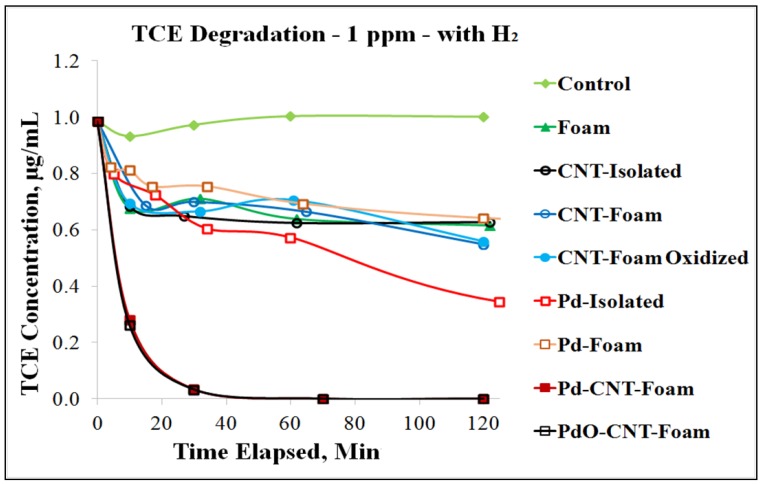
TCE degradation curves with different hierarchical elements.

Palladium (Pd) and Palladium Oxide (PdO) nanoparticles were dispersed in the hierarchical format above and found to completely remediate TCE at low concentrations (Figure 10). Also notable is that Pd on the porous carbon substrate by itself has comparable or lower degradation efficiency as isolated Pd by itself. However, Pd on the hierarchical format (attached to CNT forests on the foam) shows significantly higher degradation efficiency than all other materials and combinations tested. This demonstrates the large increase in catalytic availability through hierarchical ordering.

On a molecular scale, catalytic degradation has been correlated to adsorption energies and site availability at surfaces and interfaces. Preferred orientation of the molecules on different facets—especially the orientation and adsorption of the bridge C=C bond—has been hypothesized as being the mechanistic precursor to electron transfer and subsequent degradation. CNTs provide a unique pillar based support system for adhesion of nanoparticles with different facets, depending on size, that can be tuned for specific degradation of a chloro organic contaminant.

## 4. TCE Adsorption on CNT: Results from Molecular Modeling

To understand the relative affinity of TCE to CNTs, all-atom molecular dynamics simulations, employing the force field parameters in our previous work [69,70], were carried out on single walled carbon nanotubes (SWCNTs) and graphite sheets. The SWCNTs have a diameter of 2 nm and length between 8 and 12 nm. The graphite is composed of four graphene sheets with system size varying from 5 × 5 nm^2^ to 15 × 15 nm^2^. Simulations with a time integral of 1 fs, with equilibration of 5 ns and a minimum production run of 1 ns carried out. Initial distribution of TCE molecules on both substrates were random, and a short run of 10 ps with soft potential was done to minimize any overlaps. Both the CNT and graphite systems were fixed.

**Figure 11:**
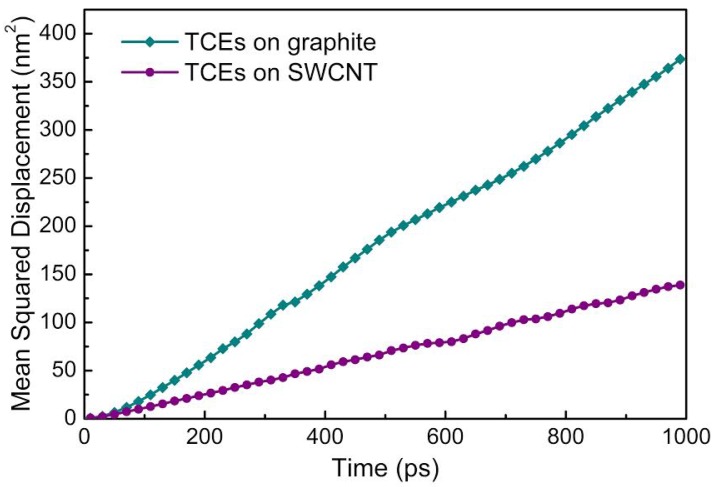
Mean-squared displacement of TCE molecules on SWCNTs and graphite at room temperature.

The mean-squared displacement (MSD) curves for both graphite and SWCNTs (Figure 11) show higher TCE mobility on the graphite surface. In the presence of water (data not shown), both mobilities decrease. For the current systems, since only van der Waals forces contribute to the adsorption of TCE on graphite and CNT, and the force fields are similar for both, curvature effects (as opposed to charge transfer and induced polarity), are expected to be the main reason for higher mobility of TCE on graphite.

The role of CNT curvature in determining the packing, orientation and adsorption of water has been examined in a number of studies, with most of the analysis focused on the inner (concave side) of the CNT [71,72,73,74]. Water structuring on the convex side [71] for graphitic surfaces is seen to be dependent on the curvature, but the hydrophobicity is independent of geometric effects. Using Monte Carlo simulations for tubular morphologies that aim to explore the effect on lateral diffusion of the curvature of lipid and protein based biological membranes [75], it was found that mean-squared displacement decreases with decrease in membrane diameter. Hindered mobility with decrease in nanoparticle size has also been seen for polystyrene chains that surround silica nanoparticles [76]. In recent work, Jana et al. [77] showed that amino acid interaction with curved graphitic interfaces is very specific to their polarity, aromaticity, and molecular size. This can also be extrapolated, as laid out by the authors [77], to analogous organic molecules. Similar observations have been made for uracil adsorption on CNTs, where flexibility of the adsorbate comes into play as the curvature of the CNT is increased [78]. Hence, CNTs present advantages in the availability of multiple surfaces (convex and concave), where the curvature can be mined for specific adsorption as well as hierarchical placement of nanoparticles. The slip behavior of both graphite and water, attributed to curvature effects, has been exploited in composite membranes, outlined in Section 2.

**Figure 12:**
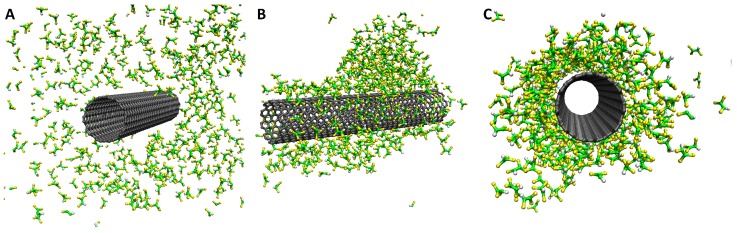
Snapshots showing adsorption of TCE on SWCNTs for the simulations carried out in the current work. A distinct monolayer forms from the (**A**) intial random to (**C**) packed conformation. There is a transition to a droplet shaped adsorption shown in (**B**).

The packing behavior of TCE molecules on the SWCNTs is shown in Figure 12. An initial random distribution goes through a droplet formation of the TCE molecules on the SWCNTs, followed by layering of the TCE molecules on the CNT surface. This intermediate droplet formation is in the absence of water and is followed by a smooth monolayer packing of the TCE. In the presence of water, Tsige et al. [69,70] have observed a droplet formation of the water molecule with TCE packing. A quantification of number and mass densities yields almost a 10% increase in the value for adsorption on SWCNTs when compared to MWCNTs [70].

**Figure 13:**
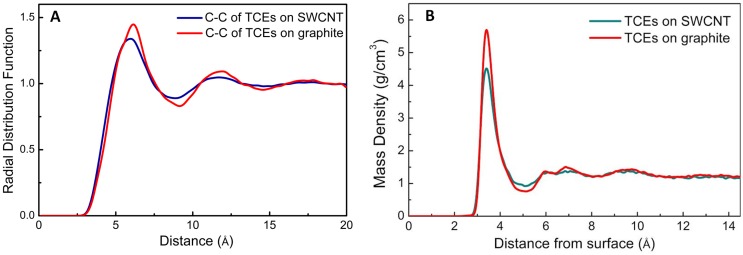
Profiles of (**A**) C-C radial distribution and (**B**) mass density for TCE adsorption on graphite and SWCNTs under study.

To compare the packing of TCE molecules on SWCNT versus graphite, radial distribution (C-C) and mass densities were computed in the current study (Figure 13). The pronounced first and second peaks in radial distribution (Figure 13A) for graphite, in comparison to SWCNTs, shows the effect of soft epitaxy leading to a more compact packing of the TCE molecules. The mass densities (Figure 13B) also show a tighter near surface packing for the TCE molecules on graphite. A similar comparative behavior for chloro organic compounds, highlighting the importance of molecular geometry and substrate effects (such as soft epitaxy), has been observed both experimentally (adsorption isotherms) [45] and theoretically (DFT computations) [79] for graphene and CNTs.

**Figure 14:**
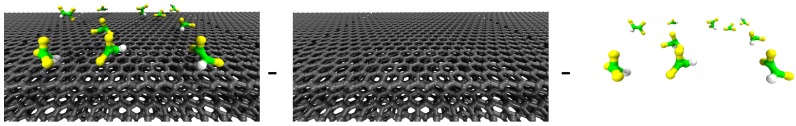
Schematic for computation of adsorption energy on graphite. The same procedure was followed for SWCNTs.

For a comparison of affinities to SWCNT and graphite, adsorption energies were computed for both systems utilizing the approach outlined in Figure 14. A total of 10 TCE molecules that would be sufficient to form a saturated monolayer with a 6 × 6 Å^2^ coordination geometry was chosen for adsorption energy calculations. The adsorption energies per TCE molecule were found to be 5.73 ± 0.35 and 8.35 ± 0.45 kcal/mol for SWCNT and graphite, respectively. The *π*-*π* interactions of the aromatic double bond with the planar graphite surface is expected to enhance the adsorption energy of TCE. It is to be noted that, in this case, the adsorption energies were only computed for the exterior surface of the CNTs. Additionally, for a range of chlorobenzene based molecules, charge transfer was found to affect SWCNT–chlorobenzenes to a larger extent when compared to graphene–chlorobenzenes, where dispersion energy was the driving force [79]. While charge transfer corrections must be applied for SWCNT adsorption, the relative role of substrate can be extracted through validated classical all-atom molecular dynamics simulations.

## 5. Conclusions & Future Outlook

The remediation of groundwater would involve competitive adsorption of natural organic matter (NOM) and a variety of contaminants on active substrates. Furthermore, change in pH, concentration, and temperature would affect design of membranes and hierarchies that can efficiently remove contaminants from groundwater. A mechanistic understanding of relative adsorption effects at the molecular level is lacking. It is expected that molecular matching to substrates through non-covalent interactions influences the adsorption and packing of a given COC as shown by Tsige et al. [69] in their work on TCB adsorption on graphite and MWCNTs, where it was found that the TCB molecule exhibited a planar conformation on graphite pointing to stacked packing. However, a more disrupted packing behavior was seen for TCB molecules on MWCNTs. The stacking of TCB on graphite would theoretically contribute to competitive adsorption between TCB and TCE in a mixture of contaminants. Competitive adsorption and the effect of chirality and pore size of CNTs for adsorption of COCs have not been studied yet, in our examination of the literature. Quantification of the interactions at play would be critical to the recursive design of composites and hybrids for water purification.

**Figure 15:**
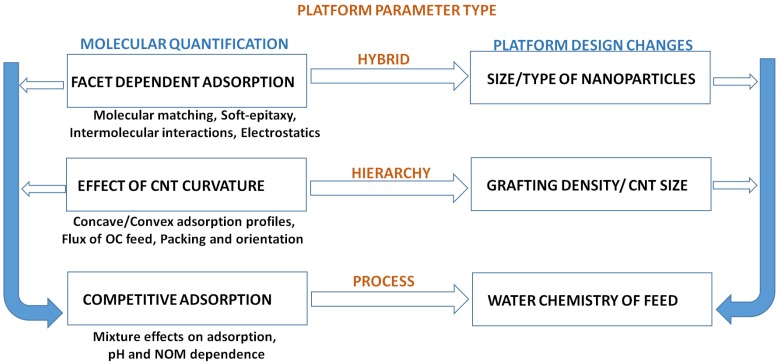
Recursive design approach to fabrication of hierarchical, hybrid remediation platforms.

Composite membranes and hierarchies, with changes in the nature of dispersed nanoparticles and fillers, allow for a combinatorial approach to remediation. To effectively harness the possibilities offered by various combinations of hybrid hierarchies towards design of highly efficient remediation platforms, the mechanistic interplay of relative interactions must be isolated for each design parameter (Figure 15). For example, using the hierarchical approach by Mukhopadhyay et al., it needs to be understood if and how the degradation of TCE and other chloro organics such as trichloro alkane may follow different pathways, depending upon water chemistry, percolation rates, nanoparticle morphologies and size distribution. With change in the size and distribution of NPs, the relative % of facets would vary. Facet specific adsorption is known to occur for organics on metal hybrids [80,81,82,83,84]. The orientation and site adsorption contours, with variation in facet distribution, would change the nature of COC adsorption (hence remediation) for a given molecular structure. Hence, COC with hierarchies would allow a high level of control in groundwater remediation for a given set of contaminants. Both adsorption on CNTs and nanoparticles for a range of COCs are currently under investigation in our research group.
